# Novel Reassortant Influenza A(H5N8) Viruses in Domestic Ducks, Eastern China

**DOI:** 10.3201/eid2008.140339

**Published:** 2014-08

**Authors:** Haibo Wu, Xiaorong Peng, Lihua Xu, Changzhong Jin, Linfang Cheng, Xiangyun Lu, Tiansheng Xie, Hangping Yao, Nanping Wu

**Affiliations:** Zhejiang University, Hangzhou, China (H. Wu, X. Peng, C. Jin, L. Cheng, X. Lu, T. Xie, H. Yao, N. Wu);; Collaborative Innovation Center for Diagnosis and Treatment of Infectious Diseases, Hangzhou (H. Wu, X. Peng, C. Jin, L. Cheng, X. Lu, T. Xie, H. Yao, N. Wu);; Zhejiang Academy of Agricultural Science, Hangzhou (L. Xu)

**Keywords:** avian influenza virus, H5N8 subtype, influenza virus, viruses, influenza, virus reassortment, domestic ducks, poultry, eastern China

## Abstract

Domestic ducks are natural reservoirs of avian influenza viruses and serve as reassortant hosts for new virus subtypes. We isolated 2 novel influenza A(H5N8) viruses from domestic ducks in eastern China, sequenced their genomes, and tested their pathogenicity in chickens and mice. Circulation of these viruses may pose health risks for humans.

Avian influenza viruses are members of the family *Orthomyxoviridae* and contain 8 segments of single-stranded RNA with negative polarity (*1*). These viruses are classified into subtypes on the basis of their envelope proteins hemagglutinin (HA) and neuraminidase (NA). Aquatic birds, including domestic ducks, have been considered the natural reservoir of these viruses (*2*). Although domestic ducks do not usually display symptoms when they are infected with these viruses, they provide an environment for the reassortment of low pathogenicity avian influenza viruses, which can serve as progenitors of highly pathogenic avian influenza viruses (*3*).

Because live poultry markets are considered a major source of avian influenza virus dissemination and sites for potential influenza virus reassortment, as well as interspecies transfer (*3*,*4*), we participated in active surveillance of these virus in live poultry markets. We sequenced genes from 2 novel influenza A(H5N8) viruses isolated from domestic ducks in eastern China and evaluated their pathogenicity in chickens and mice.

## The Study

During surveillance of poultry for avian influenza viruses in live poultry markets in Zhejiang Province in eastern China in 2013, we isolated 2 influenza A(H5N8) viruses, A/duck/Zhejiang/W24/2013(H5N8) (W24) and A/duck/Zhejiang/6D18/2013(H5N8) (6D18), from domestic ducks. To better understand genetic relatedness between these viruses, we sequenced all gene segments of these 2 viruses and compared them with influenza virus sequences in GenBank. We also determined the virulence of the 2 isolates in chickens and mice.

For virus isolation, cloacal swab specimens from domestic ducks were inoculated into embryonated chicken eggs as described (*5*). All experiments with viruses were performed in a Biosafety Level 3 laboratory.

RNA was extracted by using the Viral RNA Mini Kit (QIAGEN, Hilden, Germany) according to the manufacturer’s instructions. All gene segments were amplified with primers, and fragments were sequenced and analyzed as described (*6*–*8*). Sequence data obtained were submitted to GenBank under accession nos. KJ476663–KJ476678.

**Figure 1:**
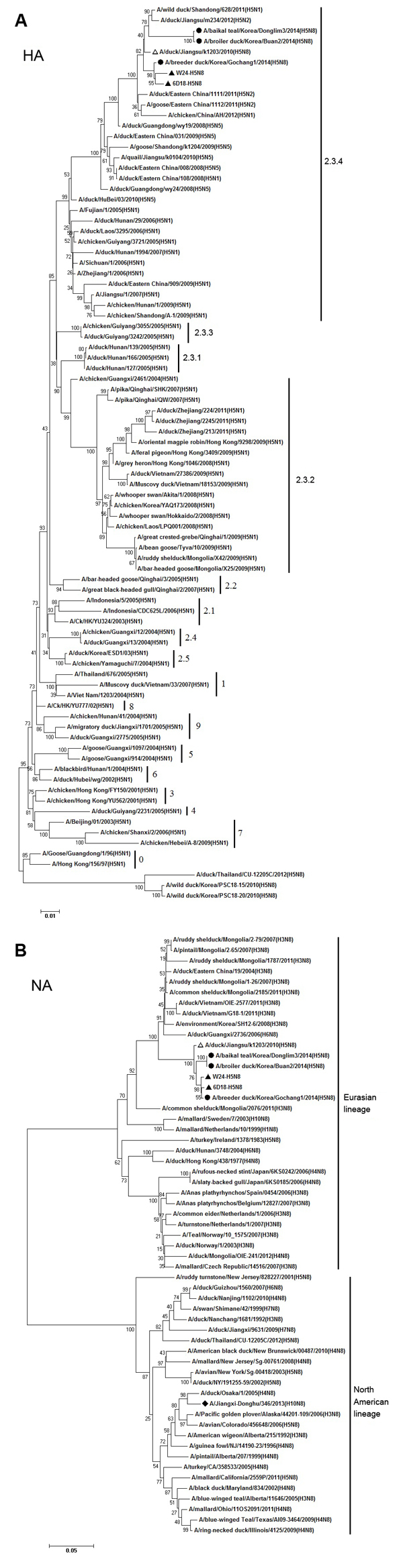
Phylogenetic analysis of A) hemagglutinin (HA) (nucleotide positions 9–1647) and B) neuraminidase (NA) (nucleotide positions 44–1381) genes of novel influenza A(H5N8) viruses isolated from domestic ducks, eastern China, 2013, and other influenza viruses. The tree was created by the using the neighbor-joining method and bootstrapped with 1,000 replicates by using MEGA 5.05 (http://www.megasoftware.net/). Influenza A(H5N8) viruses from China identified in this study are indicated by solid triangles, another influenza A(H5N8) virus from China is indicated by a white triangle, 3 influenza A(H5N8) viruses from South Korea (2014) are indicated by dots, and a novel influenza A(H10N8) virus (2013) that caused human infection is indicated by a diamond. Scale bars indicate distance units between sequence pairs.

Sequence analysis showed that all sequences of 8 genes (HA, NA, basic polymerase 1, basic polymerase 2 [PB2], acidic polymerase, nucleoprotein, matrix protein, and nonstructural protein [NS]) of viruses W24 and 6D18 showed 99.9%–100% sequence similarity (Figure 1). Results show that the HA gene of W24 was closely related to those HA genes of H5N8 subtype viruses circulating in South Korea in 2014 (*9*); W24 belongs to clade 2.3.4.

Sequence analysis suggested that these H5N8 subtype viruses were most closely related to isolates from poultry in countries in eastern Asia. Previous studies have shown that H5 subtype viruses within clade 2.3.2 have been circulating widely in poultry and wild birds in China since 2007 (*7*,*10*). Our results indicated that the 2 novel H5 subytpe viruses belong to clade 2.3.4, the prevalent lineage in southern China since 2005 (*11*); thus showing their presence in eastern China. NA gene phylogeny indicated that a novel influenza A(H10N8) virus, which infected humans, had different ancestors for this gene (Figure 1, panel B).Gene phylogenies for 6 other genes indicated that H9N2 subtype viruses circulating in China were not donors of these genes for W24.

**Figure 2:**
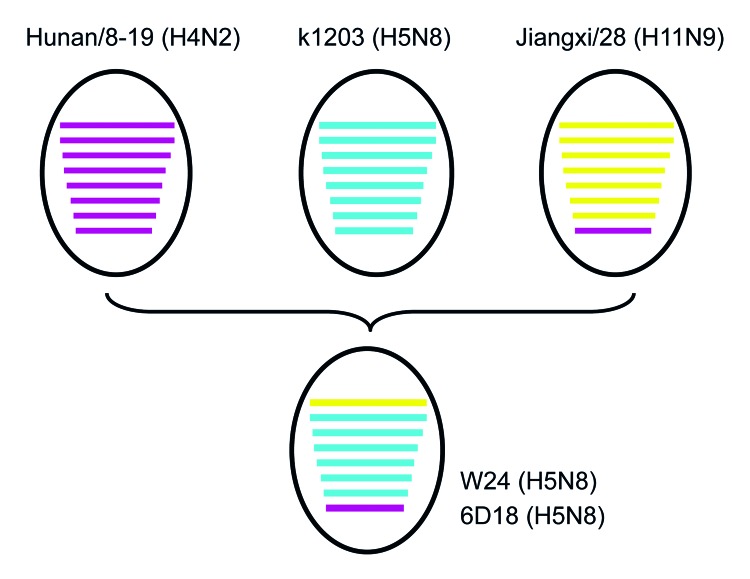
Putative genomic compositions of novel influenza A(H5N8) viruses isolated from poultry, eastern China, 2013, and their 3 possible parent viruses. The 8 gene segments (from top to bottom) in each virus are basic polymerase 2, basic polymerase 1, acidic polymerase, hemagglutinin, nucleoprotein, neuramindase, matrix, and nonstructural protein. Each color represents a separate virus background: purple indicates Hunan/8-19 (H4N2); A/duck/Hunan/8–19/2009(H4N2); blue indicates k1203 (H5N8), A/duck/Jiangsu/k1203/2010(H5N8); and yellow indicates Jiangxi/28 (H11N9), A/environment/Jiangxi/28/2009(H11N9). The simplified schematic illustration is based on nucleotide-distance comparison and phylogenetic analysis.

Lee et al. (*9*) recently reported that that HA and NA genes of 3 H5N8 subtype viruses isolated in South Korea in 2014 had high nucleotide identities with A/duck/Jiangsu/k1203/2010(H5N8). All 8 genes of W24 were closely related to those of H5N8 subtype viruses, such as A/breeder duck/ Korea/Gochang1/2014(H5N8), which are circulating in South Korea. Basic polymerase 1, acidic polymerase, HA, nucleoprotein, NA, and matrix genes of W24 were also closely related to those of A/duck/Jiangsu/k1203/2010(H5N8). PB2 and NS genes of W24 were most closely related to those of A/environment/Jiangxi/28/2009(H11N9) and A/duck/Hunan/8–19/2009(H4N2), respectively (Table 1; Figure 2). On the basis of analysis of phylogenetic relationships, we found that W24 was a reassortant virus that derived its genes from a virus of a different subtype from poultry in China. We also found that H5N8 subtype viruses had been present in eastern China for several years and these viruses might have been spread to other countries by wild birds in recent years.

**Table 1:** Sequence homology of whole genome of influenza A(H5N8) A/duck/Zhejiang/W24/2013 virus isolated from domestic ducks, eastern China, 2013, compared with nucleotide sequences available in GenBank*

Gene	Virus with the highest percentage of nucleotide identity	GenBank accession no.	Homology, %
PB2	A/breeder duck/Korea/Gochang1/2014 (H5N8)	KJ413831	99
	A/environment/Jiangxi/28/2009 (H11N9)	KC881295	98
PB1	A/breeder duck/Korea/Gochang1/2014 (H5N8)	KJ413832	99
	A/duck/Jiangsu/k1203/2010 (H5N8)	JQ973692	99
PA	A/breeder duck/Korea/Gochang1/2014 (H5N8)	KJ413833	99
	A/duck/Jiangsu/k1203/2010 (H5N8)	JQ973693	99
HA	A/breeder duck/Korea/Gochang1/2014 (H5N8)	KJ413834	99
	A/duck/Jiangsu/k1203/2010 (H5N8)	JQ973694	99
NP	A/breeder duck/Korea/Gochang1/2014 (H5N8)	KJ413835	99
	A/duck/Jiangsu/k1203/2010 (H5N8)	JQ973695	99
NA	A/breeder duck/Korea/Gochang1/2014 (H5N8)	KJ413836	99
	A/duck/Jiangsu/k1203/2010 (H5N8)	JQ973696	99
M	A/breeder duck/Korea/Gochang1/2014 (H5N8)	KJ413837	99
	A/duck/Jiangsu/k1203/2010 (H5N8)	JQ973697	99
NS	A/breeder duck/Korea/Gochang1/2014 (H5N8)	KJ413838	99
	A/duck/Hunan/8–19/2009 (H4N2)	HQ285890	99

*PB2, basic polymerase 2; PB1, basic polymerase 1; PA, acidic polymerase; HA, hemagglutinin; NP, nucleoprotein; NA, neuraminidase; M, matrix; NS, nonstructural protein.

On the basis of deduced amino acid sequences of HA genes, we found that the HA cleavage site pattern (PLREKRRKR) of the 2 novel H5N8 subtype viruses indicated that these viruses were highly pathogenic. In this study, amino acid sequences of these H5N8 subtype viruses at positions 236–241 and 146–150 were NGQRGR and GVSAA, respectively. Receptor-binding sites (Gln226 and Gly228) of H5N8 subtype viruses were similar to those of the 2 novel H5N8 subtype viruses, which suggested that these 2 viruses would preferentially bind to avian-like receptors (*7*). The PB2 protein Lys627Glu mutation has been reported to influence the host range and confer increased virulence for H5N1 subtype viruses in animal models (*12*). This mutation was not observed in PB2 of the 2 novel H5N8 subtype viruses analyzed in this study, which indicated that these 2 viruses had low levels of pathogenicity for mice.

Deletion of several amino acids (position 80–84) in NS1 proteins had been observed more frequently in H5N1 subtype viruses, which indicated possible adaptation of these viruses to avian species (*13*). This deletion was not observed in the 2 novel H5N8 subtype viruses. These 2 viruses contained the NS1 Pro42Ser mutation, which is associated with increased virulence in mice (*14*).

To evaluate pathogenicity of W24 and 6D18 in chickens, we inoculated groups of ten 6-week-old specific pathogen–free chickens intravenously with a 10^6^ median egg infective dose of each virus in a 0.2-mL volume of phosphate-buffered saline; deaths were observed over a 10-day period (*7*). Animal studies were conducted according to the recommendation of the World Organisation for Animal Health (Paris, France). Characteristics of W24 and 6D18 viruses are shown in Table 2. Results showed that these viruses were highly pathogenic to chickens.

**Table 2:** Characteristics of 2 novel influenza A(H5N8) viruses isolated from domestic ducks, eastern China, 2013 *

Virus	Characteristic			Virus replication in experimentally infected mice, virus titers in organs of mice (log_10_ EID_50/_mL)†			
	IVPI	EID_50_	TCID_50_	Tissue	3 d	6 d	9 d
W24	3.0	10^8.5^	10^8.4^	Lung	2.5 ± 0.5	3.0 ± 0.5	5.0 ± 0.5
	NA	NA	NA	Brain	–	–	–
	NA	NA	NA		–	–	–
	NA	NA	NA	Liver	–	–	–
6D18	3.0	10^8.5^	10^9.6^	Lung	2.0 ± 0.5	2.5 ± 0.5	3.0 ± 0.5
	NA	NA	NA	Brain	–	–	–
	NA	NA	NA	Heart	–	–	–
	NA	NA	NA	Liver	–	–	2.0 ± 0.5

*Groups of 14 adult female BALB/c mice were intranasally inoculated with a 10^6^ median egg infective dose (EID_50_) of each virus in a 0.05-mL volume of phosphate-buffered saline. Five mice per group were observed for survival within 14 d. The remaining 9 mice from each experimental group were exsanguinated on days 3, 6, and 9, and their lungs, brains, hearts and livers were collected for virus titration in embryonated chicken eggs. IVPI, intravenous pathogenicity index; TCID_50_, median tissue culture infective dose; NA, not applicable.  †Values are mean ± SD. –, virus was not detected.

To determine the pathogenicity of these viruses in a mammalian host, we inoculated BALB/c mice intranasally with a 10^6^ median egg infective dose for each virus. Over a 14-day period, we observed virus replication in various organs and deaths. After intranasal administration of W24 and 6D18, we observed 5 mice per group for survival over a 14-day period. On day 9 postinfection, high titers of virus was detected in lung and liver but were not detected in brain and heart. Mice had signs of illness but had survival rates of 80% (4/5) and 100% (5/5), respectively, for each virus during 14 days postinfection (Table 2). These results suggested that the 2 novel H5N8 subtype viruses did not kill mice but that they could replicate in the lung. Results of our study are consistent with those of Zhao et al. (*15*), who reported that H5N8 subtype virus from chickens did not cause deaths in mice.

## Conclusions

We isolated 2 influenza A(H5N8) viruses were isolated from domestic ducks in eastern China in 2013. Results of phylogenetic analysis showed that these viruses were reassortant viruses that derived their genes from different virus subtypes. These reassortant H5N8 subtype viruses and their 3 possible parent viruses, A/duck/Jiangsu/k1203/2010 (H5N8), A/environment/Jiangxi/28/2009 (H11N9), and A/duck/Hunan/8–19/2009 (H4N2), were isolated in China. Both H5N8 subtype isolates were highly pathogenic for chickens and showed moderate pathogenicity for mice. Domestic ducks are considered the natural reservoir of avian influenza viruses and serve as reassortant hosts for creation of new virus subtypes. Continued circulation of these viruses may pose health threats for birds and humans. 
